# Early weight bearing in tibial plateau fractures treated with ORIF: a systematic review of literature

**DOI:** 10.1186/s13018-022-03156-8

**Published:** 2022-05-12

**Authors:** Gianluca Canton, Andrea Sborgia, Micol Dussi, Nicholas Rasio, Luigi Murena

**Affiliations:** Orthopaedics and Traumatology Unit, Department of Medical, Surgical and Life Sciences, Cattinara Hospital, Trieste University, Strada di Fiume 447, 34149 Trieste, Italy

**Keywords:** Early weight bearing, EWB, Tibial plateau fracture, Internal fixation, ORIF

## Abstract

**Background:**

To review the current clinical evidence on advantages and risks of early weight bearing (EWB) after internal fixation for tibial plateau fracture.

**Methods:**

Data source: PubMed and Google Scholar from inception of database to 20 August 2021**,** using PRISMA guidelines. The included studies were randomized controlled trials, prospective and retrospective observational studies, case reports. Data extraction was performed independently by 2 reviewers. Collected data were compared to verify agreement. Statistical analysis was not performed in this study.

**Results:**

The literature search produced 174 papers from PubMed and 186 from Google Scholar, with a total amount of 360 papers. The two reviewers excluded 301 papers by title or duplicates. Of the 59 remaining, 33 were excluded after reading the abstract, and 17 by reading the full text. Thus, 9 papers were finally included in the review.

**Conclusions:**

EWB can be considered safe and effective in selected cases after internal fixation for tibial plateau fractures.

*Level of evidence* Therapeutic Level III.

**Supplementary Information:**

The online version contains supplementary material available at 10.1186/s13018-022-03156-8.

## Introduction

Tibial plateau fractures represent approximately 1.2% of all fractures in the adult population [1], with an incidence of 13.3 per 100,000 per year [2]. These fractures include a wide range of different patterns, from simple configurations to very complex multifragmentary fractures, with various degrees of displacement and articular depression [3]. Even if there is no consensus on the best method of fixation for these fractures [4], open reduction internal fixation (ORIF) is the mostly used technique. The goal is to obtain anatomic reduction of the articular surface, lower limb axis alignment and knee joint stability. The impact of these fractures on patients’ lives and health care system is widely described in literature. In many literature reports, patients cannot return to work before 3–4 month after surgery [5] and only 39% of patients return to work within one year [6]. Percentage of patients who return to sport [7] is lower in patients with tibial plateau fractures compared to other lower limb fractures and knee stiffness is reported to range between 3 and 18% [8–10]. Post-operative rehabilitation is a key step to obtain satisfactory results. However, there is a lack of evidences and consensus about the best postoperative rehabilitation, especially concerning recommended postoperative weight bearing. Orthopedic surgeons usually prefer to restrict weight bearing after tibial plateau fracture fixation, in order to reduce the risk of loss of reduction and implant failure. According to Arbeitsgemeinschaft für Osteosynthesefragen (AO) classic principles of fracture fixation, no weight bearing or restricted (toe touch) weight bearing for 6–8 weeks to 10–12 weeks should be the standard of care for tibial plateau fractures treated with ORIF [11]. However, recent literature reports suggest that early weight bearing post-operative protocols could be applied to selected tibial plateau fracture cases after internal fixation, with low rates of mechanical complications, a faster return to work and better clinical outcomes [12]. Aim of the present paper is to review the current clinical evidence on advantages and risks of early weight bearing (EWB) protocols after internal fixation for tibial plateau fracture.

## Materials and methods

Two reviewers (MD and AS) independently identified studies by a systematic search of PubMed and Google Scholar from inception of database to 20 August 2021. The search strategy was structured according to the PICO (Population Intervention Comparison Outcome) method as follows: P “(tibial plateau fracture ORIF”) AND I (“early weight bearing” OR “immediate weight bearing” OR “postoperative weight bearing”) AND O (“loss reduction” OR “rehabilitation” OR “return to work”). The two reviewers screened the titles and abstracts of the citations identified independently and in duplicate, and acquired the full text of any article that either judged potentially eligible. The inclusion criteria for the studies were: (a) English written papers, (b) all studies concerning early or immediate weight bearing in articular and/or partial articular tibial plateau fracture treated with ORIF, (c) all article types including case reports. The exclusion criteria were: (a) language of publication different from English (b) tibial plateau fractures treated non operatively, (c) tibial plateau fractures treated with external fixation, (d) extra-articular proximal tibia fractures, (e) animal research and biomechanical models, (f) book chapters.

**Fig. 1:**
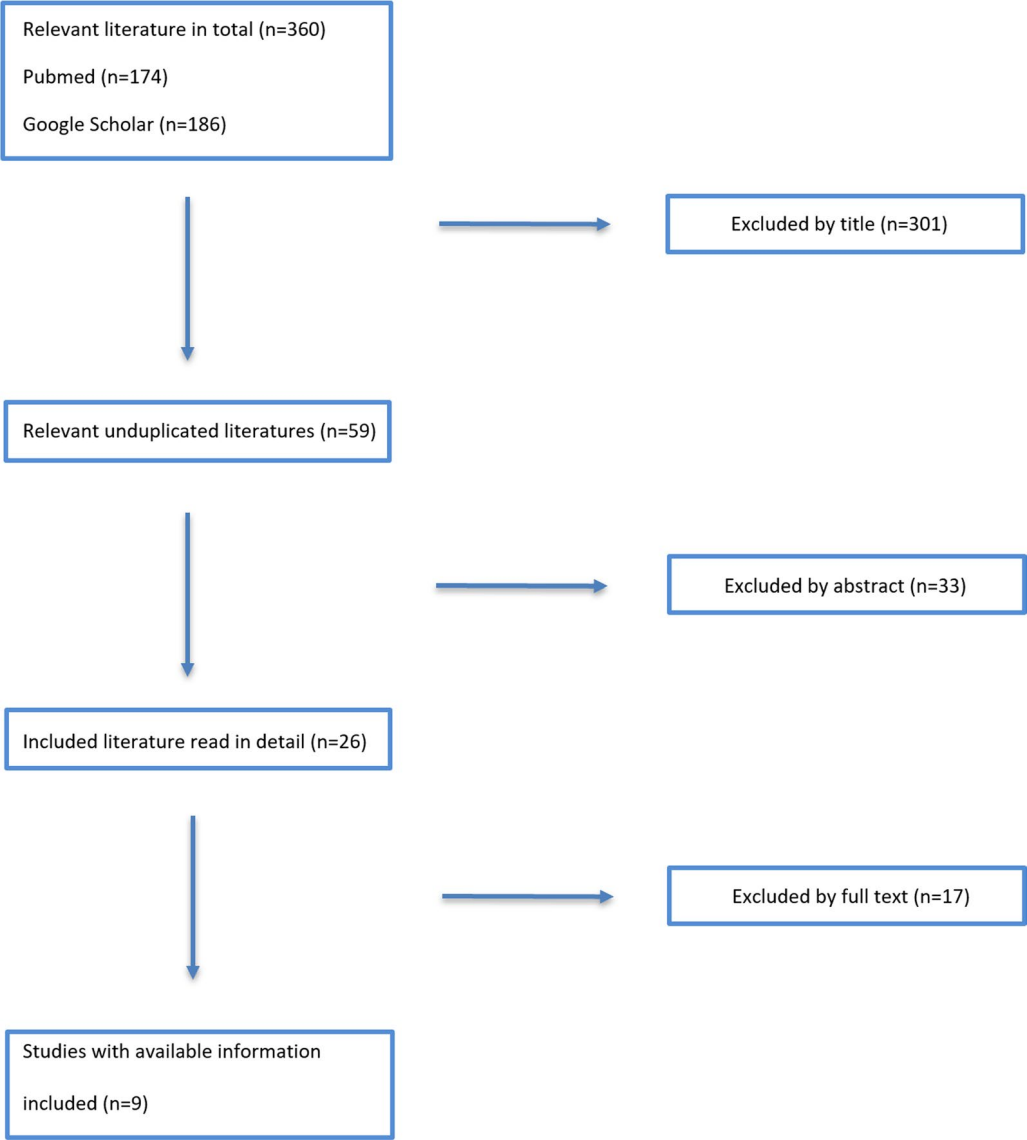
PRISMA flowchart

The present review was conducted in accordance with the 2009 Preferred Reporting Items for Systematic Review and Meta-Analysis (PRISMA) statement (Fig. 1—PRISMA flowchart) (Additional file 1: PRISMA checklist).

### Risk of bias assessment

Risk of bias was assessed on all the included studies in the quantitative analysis based on OHAT Risk of Bias Rating Tool for Human and Animal Studies. Domains were defined as selection bias, confounding bias, attrition/exclusion bias, detection bias and selective reporting bias and were rated as “*Definitely Low risk*”, “*Probably Low risk*”, “*Probably High risk*”, or “*Definitely High risk*” at the level of interest of population and outcome for this systematic review. Two co-authors independently assessed the risk of bias (AS and MD), and disagreement was resolved by consensus and discussed with another author (GC).

## Results

The literature search produced 174 papers from PubMed and 186 from Google Scholar, with a total.

amount of 360 papers. The two reviewers excluded 301 papers by title or duplicates. Of the 59 remaining, 33 were excluded after reading the abstract, and 17 by reading the full text. Thus, 9 papers were finally included in the review. Details of studies are described in Table 1.

**Table 1:** Characteristics of included studies

Study	Patients	Age (mean)	Classification	FU	NWB/TTWB	EWB	Amount EWB	Complications				
								Articular collapse/joint depression in EBW group	Amount of articular collapse/joint depression	Implant failure in EBW group	Fracture fragments migration in EBW group	Non union in EBW group
Williamsson et al.	90	45.1	Schatzker type II	3 months	60	30	As tolerated	1	4 mm	–	NE	NE
Haak et al.	32	48	AO Type B	6–8 weeks	20	12	As tolerated	–	–	–	NE	NE
Thewlis et al.	17	52	Schatzker type from II to VI	52 weeks	–	17	20 kg	–	–	–	NE	NE
Eggli et al.	14	41	AO type C	1 year	–	14	10 kg	–	–	–	NE	–
Thewlis et al.	9	51.4	Schatzker type II–III–IV	52	–	9	As tolerated	NE	NE	–	< 3 mm	NE
Solomon et al.	7	39	Schatzker type II	52 weeks	–	7	20 kg	NE	NE	NE	< 1 mm	NE
Kalmet et al.	91	50.8	Schatzker all type	1 year	60	31	Permissive	–	–	–	NE	1
Hare et al.	1	44	AO type B	1 year	–	1	As tolerated	–	–	–	NE	NE
Callary et al.	143	45–48	AO type B–C	2 years	–	143	As tolerated	–	–	–	–	NE

*EWB* early weight bearing; *TTWB* toe touch weight bearing; *NWB* no weight bearing; *FU* follow-up

### Risk of bias assessment

The risk of selection bias was “*Definitively Low*” in 7 studies (77%), “*Probably Low risk*” for one study (11%) and not applicable for the case report. The risk of confounding bias was “*Probably High risk*” in 5 studies (55%) and “*Probably Low risk*” in 4 studies (44%). The risk of attrition/exclusion bias was “*Definitively Low*” in 2 studies (22%) and “*Probably Low risk*” in 6 studies (66%, while was not applicable for the case report). The risk of detection bias was “*Probably Low risk*” in 4 studies (44%), “*Probably High risk*” in 4 studies (44%) and “*Definitely High risk*” in 1 study (11%). Reporting bias was “*Definitively Low*” for 3 studies and “*Probably Low risk*” for 6 studies (66%) (Table 2).

**Table 2:**
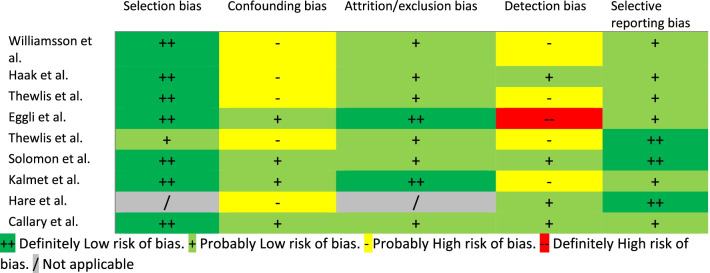
OHAT risk of bias rating tool

*Selection bias* refers to systematic differences between baseline characteristics of the groups that are compared; *Counfunding bias* relating to confounding and co-exposures is addressed under selection bias and performance in study quality tools such as Cochrane, AHRQ, and SYRCLE; *Attrition or exclusion bias* refers to systematic differences in the loss or exclusion from analyses of participants or animals from the study and how they were accounted for in the results; *Detection bias* refers to systematic differences between experimental and control groups with regards to how outcomes and exposures are assessed and also considers validity and reliability of methods used to assess outcomes and exposures; *Selective reporting bias* refers to selective inclusion of outcomes in the publication of the study on the basis of the results.

## Discussion

**Fig. 2:**
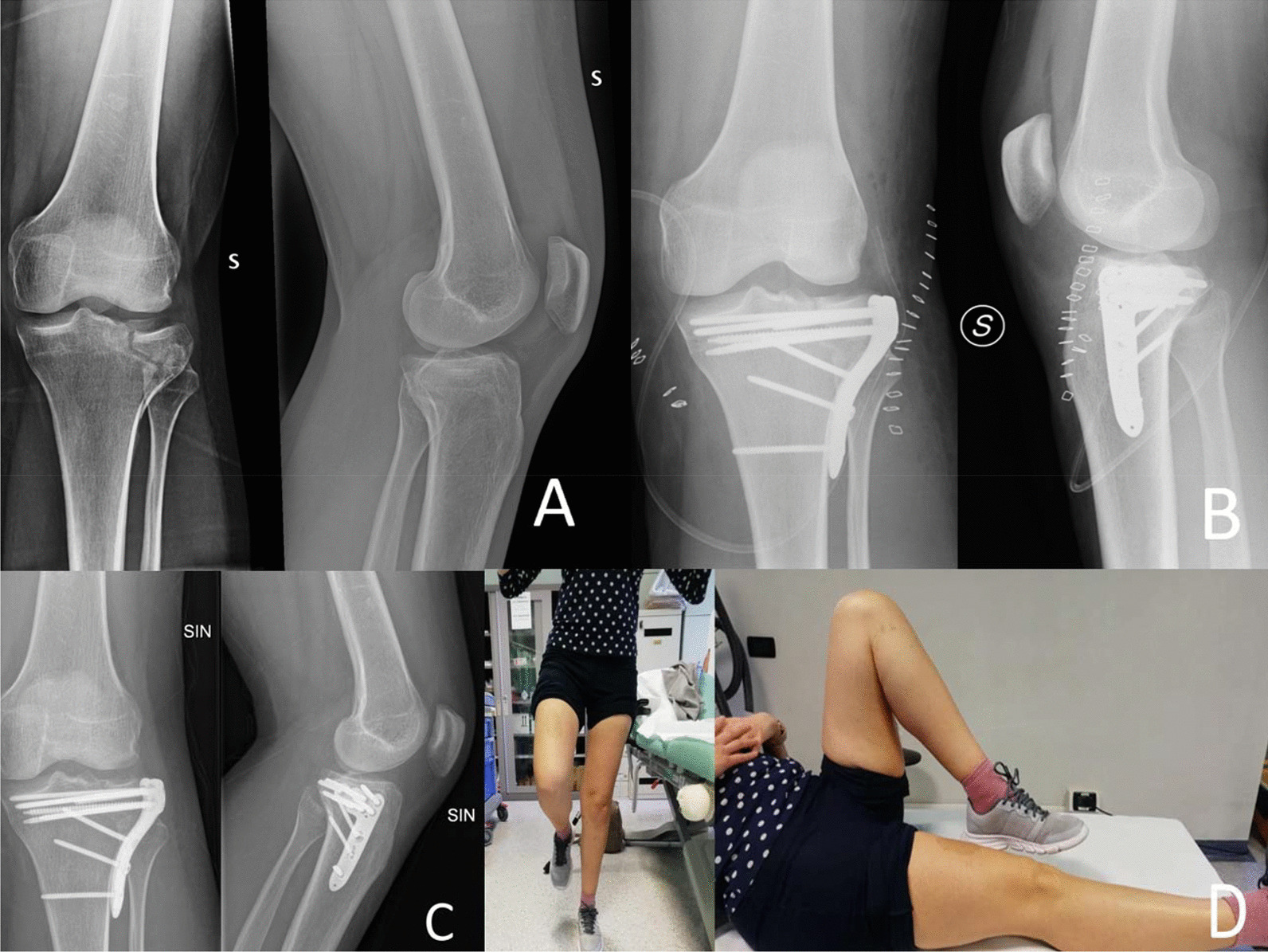
Tibial plateau fracture Schatzker II treated with ORIF followed by post‐operative EWB protocol: 20 kg for the first 7 days, then weight bearing as tolerated. **A** Preoperative X‐rays. **B** Post‐operative X‐rays. **C** X‐rays at 5 weeks. **D** Clinical outcome at 5 weeks

The risk of reduction loss, especially in complex articular fractures, is a concern for the orthopedic surgeon. Therefore, patients treated with ORIF for tibial plateau fractures usually observe a variable period of restricted weight bearing. However, there is a lack of consensus on post-operative management for these patients. Despite non weight-bearing protocols may prevent loss of reduction and articular surface collapse, EWB may have consistent benefits in such patients, especially in terms of lower energy expenditure during deambulation [13], faster recovery of full weight bearing, faster return to work and sport [7] and shorter hospitalization [14]. Nonetheless, there must be a balance between the benefits of EWB and the risks of mechanical complications. The papers retrieved in the present review seem to sustain this hypothesis (Fig. 2).

The main concern regarding weight bearing in tibial plateau fractures is the loss of reduction. Most studies in fact focus on this topic, and although different rehabilitation protocols were used, none of the studies analyzed reported loss of reduction after early postoperative weight bearing.

Williamsson et al., in a retrospective-prospective study involving 90 patients (mean age 45.1 ± 16.5) with tibial plateau fractures treated with ORIF, divided the patients in two groups: the first one consisted of 60 patients with no weight-bearing or toe-touch weight-bearing for 6 postoperative weeks, the second one (30 patients) was allowed immediate weight bearing as tolerated. 51% of the patients had a Schatzker II tibial plateau fracture and the remainder were Schatzker I, IV, and VI fracture patterns. All patients had postoperative radiographs at first or second day post-surgery and after six weeks and three months. Only one patient of the weight bearing group had > 1 mm (4 mm) joint depression on the two follow up radiographs, with no worse clinical or functional outcome compared to the other patients. The author concluded that immediate weight bearing was not related with articular collapse or fixation failure and that patients with tibial plateau fractures should be allowed immediate full weight bearing on the operated limb after fracture fixation [15].

In a retrospective study performed by Haak and collegues, involving 32 patients with partial articular tibial plateau fractures (AO type 41B) treated with locking plate osteosynthesis, the authors divided the patients in two groups: the first group (20 patients; mean age 51.5) had indication for no weight bearing on the operated limb, the second (12 patients; mean age 43.5) was allowed immediate postoperative weight bearing. No radiographic fracture displacement or implant failure was reported in any patient even in the last 6–8 weeks follow-up. The author concluded that allowing immediate weight bearing in partial articular tibial fractures treated by osteosynthesis with locking plate was possible and safe [16].

Similarly, in a study conducted by Thewlis and colleagues involving 17 patients with tibial plateau fractures treated with ORIF (AO Type B-C), patients were prescribed isometric quadriceps exercise (before and after surgery), immediate postoperative full knee range of motion and weight bearing on the operated limb up to 20 kg for the first six weeks. Dynamic weight bearing in order to assess a ratio of limb loading (operated vs non operated) was conducted at 2, 6 and 12 weeks after surgery, Knee Injury and Osteoarthritis Outcome Scores (KOOS) were collected at 6, 12, 26, and 52 weeks after surgery and all patients were analyzed with plain radiographs immediately post-surgery and at 52 weeks. Fracture fragments’ migration or changes in knee stability and alignment was not noted in any patient at 52 weeks and the dynamic weight bearing performed at 2 and 6 weeks post-surgery demonstrated no correlation with knee-related Quality of Life at 52 weeks [17]. The authors concluded that early weight bearing was safe and was not associated with implant failure or loss of reduction.

Eggli and colleagues performed a prospective study on 14 patients (mean age 41 ± 27) with bycondilar tibial plateau fracture (AO type C) treated with double plating, with the addition of autologous cancellous iliac crest graft in 11 cases. All patients had preoperative antero-posterior (AP) and lateral view x-rays and computed tomography (CT) scans. The patients were evaluated at 8, 12, and 24 weeks, and at 1 year after surgery with clinical exam and both AP and lateral views. All patients were allowed immediate 10 kg weight bearing on the operated limb. All patients achieved union within 12 weeks and no patient experienced loss of fracture reduction [18].

Thewlis and colleagues performed a prospective longitudinal study involving 9 patients with partial articular tibial plateau fractures (AO type B) treated with ORIF, allowing immediate weight bearing as tolerated. The mean age of all patients was 51.4. The aim of the study was to quantify the migration of bony fragments and the loads passing through the fracture construct using radioesterometric analysis. The patients were evaluated at 2, 12, 26, and 52 weeks after surgery. CT scans were taken postoperative and at one-year follow-up to assess maintenance of fracture reduction. Fracture migration was measured as the motion of the center of the fracture fragment relative to the center of the unfractured tibial plateau. Migration was defined acceptable if the articular surface subsided ≤ 3 mm during healing). The authors found that whilst peak loading during walking was not associated with fracture migration, average force, exercised during the stance phase of gait, was majorly correlated with fragment migration. Despite this finding migration was not higher than 3 mm in none of the patients [19].

Solomon et al. described 7 Schatzker type II tibial plateau fractures (two women and five men, with a mean age of 39 years) treated with subcondral screws and buttress plate. All patients were allowed partial weight bearing up to 20 kg using crutches for six weeks. In addition to traditional radiographs, radiostereometric analysis (RSA) and differentially loaded radiostereometric analysis (DLRSA) were performed at 2, 6, 12, 18, 26, and 52 weeks. Displacement of fractures fragments was < 1 mm in all patients, in both craniocaudal and mediolateral direction. The mean fracture fragment displacement in craniocaudal direction was −0.30 mm (−0.73 to 0.02) and 0.00 mm (−0.12 to 0.15) at 2 and 52 weeks respectively. The mean displacement in mediolateral direction was −0.08 (−0.73 to 0.36) and −0.01 (−0.16 to 0.11) at 2 and 52 respectively [20].

As stated, faster recovery of full weight bearing and faster return to work and sport [7] might also be potential advantages of EWB protocols after tibial plateau fractures fixation.

Kalmet et al. evaluated a series of 91 patients with tibial plateau fractures in a retrospective cohort study with the aim to identify the differences in term of quality of life, pain and complications between patients with tibial plateau fractures with postoperative indication of permissive weight bearing (PWB, 31 patients) and patients with restricted weight bearing (RWB, 60 patients). In the PWB group patients had significantly (*p* = 0.04) more severe fracture patterns (Schatzker type IV–VI). There were no statistically significant differences in terms of quality of life, pain and complication rates between the two groups, but patients with PWB reached full weight bearing earlier (14.7 weeks vs 20.7 weeks, *p* = 0.02) and the number of patients who reached full weight bearing within 12 weeks was significantly higher in the PWB group (58.1% vs 28.3%, *p* < 0.01). The authors concluded that permissive weight bearing after tibial plateau fracture was safe and lead to a significant reduction in time to reach full weight bearing without differences in terms of complications, pain and quality of life [21].

Callary et al. included in their study 143 patients (145 fractures) with tibial plateau fracture (AO type B-C) and divided them into 2 groups. In the first group (“Classic group”) the patients were treated with plate and screws according to the standard surgical technique. In the second group the patients were instead treated with a different approach, preserving the perforating vessels: Angiosome of Perforator-Sparing (APS). In both groups after surgery the patients were allowed full weight bearing and were encouraged to mobilize as early as possible routine preoperative CT scans were performed for all patients while postoperatively CT is only requested when quality of reduction is unclear on radiographs. The authors found no significant increase in wound complications or fixation failure in the second group compared to the “classic group”, despite the earlier and more aggressive rehabilitation process [22].

In the literature few studies evaluate time to return to work and sport. Furthermore, the few studies considered did not associate rehabilitation protocols with EWB and didn’t compare the results with a delayed weight bearing control group. We found a case report of a 44 year old man with a lateral tibial plateau fracture (AO 41B2) diagnosed with CT scans and treated with highly impacted bone allograft and angular stable plate fixation. The patient was allowed immediate weight bearing on the affected limb and follow up was performed at 2 and 6 weeks and 3 and 12 months after surgery. There was no widening of the tibial plateau, no collapse of the articular surface, the patient at 6 weeks had 0–125 degrees of range of motion (ROM) and returned to work three months after surgery [23].

Robertson et al. [7] in their review report that patients sustaining a tibial plateau fracture have lower return to sport rates compared to other types of injury but all the studies concerning tibial plateau fractures treated with ORIF (*n* = 193) have a variable time of delayed weight bearing and do not consider EWB as a rehabilitation protocol. There is, therefore, lack of data on average time to return to work and sport.

The retrieved papers also allow to understand the key role of early rehabilitation, which has a well-known role in recovery after fracture fixation and gains even more importance in association with EWB protocols. In all studies analyzed, patients returned to normal gait and early knee ROM without evidence of complications.

However, the retrieved studies often did not give sufficient information about post-operative rehabilitation programs and when described there was a wide disparity of indications. In most studies early weight bearing seems to be reserved to young patients with simple fracture patterns (Schatzker type II; AO type B, but other important limitations concerning EWB literature should be emphasized. None of the studies evaluated potential results modifiers as body mass index (BMI), knee function before trauma and associated of intra-articular lesions. Furthermore, only in a few studies patients are divided according to fracture pattern and it is therefore difficult to establish the most suitable post-operative rehabilitation protocol for tibial plateau fractures based on the severity of the fracture pattern.

## Conclusions

Early weight bearing in tibial plateau fractures treated with internal fixation has shown comparable complications in terms of loss of reduction, mechanical failure and delayed healing with respect to restricted weight bearing protocols. The retrieved studies do not allow to draw definitive conclusions on time to return to work and sport activities, as well as greater autonomy in daily life activities and better motility. To accurately evaluate the safety of EWB we will need longer clinical trials with a greater number of patients and that more clearly define the indications or limitations of early weight bearing protocols.

## Supplementary Information

**Additional file 1.**PRISMA 2020 Checklist.

## Data Availability

All data generated or analysed during this study are included in this published article.

## Abbreviations

ORIF: Open reduction internal fixation
AO: Arbeitsgemeinschaft für Osteosynthesefragen
EWB: Early weight bearing
KOOS: Knee Injury and Osteoarthritis Outcome score
RSA: Radiostereometric analysis
DLRSA: And differentially loaded radiostereometric analysis
PWB: Permissive weight bearing
RWB: Restricted weight bearing
ROM: Range of motion
BMI: Body mass index
